# α,β-Pipitzols and α,β-Isopipitzols from Natural Quinone Perezone: Quantum Chemistry, Docking, Chemoinformatic, and Pharmacological Studies

**DOI:** 10.3390/molecules31030469

**Published:** 2026-01-29

**Authors:** Adriana Lizbeth Rivera Espejel, Joel Martínez, Cristopher Williams Fuentes Cid, Martha E. Macías Pérez, Maricarmen Hernández Rodríguez, Alejandro Fajardo De La Rosa, René Miranda Ruvalcaba, María Inés Nicolás-Vázquez

**Affiliations:** 1 Departamento de Ciencias Químicas, Facultad de Estudios Superiores Cuautitlán Campo 1, Universidad Nacional Autónoma de México, Avenida 1° de Mayo s/n, Colonia Santa María las Torres, Cuautitlán Izcalli 54740, Mexico; riveraespejeladriana@gmail.com (A.L.R.E.); atlanta126@gmail.com (J.M.); 316048734@cuautitlan.unam.mx (C.W.F.C.); 2 Unidad de Investigación Biomédica de Zacatecas (UIBMZ) del Instituto Mexicano del Seguro Social (IMSS), Alameda Trinidad García de La Cadena 438_2436A436, Zacatecas Centro, Zacatecas 98000, Mexico; marthita_e23@yahoo.com.mx; 3 Laboratorio de Cultivo Celular, Escuela Superior de Medicina, Instituto Politécnico Nacional (IPN), Salvador Díaz Mirón esq. Plan de San Luis s/n, Casco de Santo Tomás, Miguel Hidalgo, Ciudad de México 11340, Mexico; dra.hernandez.ipn@gmail.com; 4 Laboratorio de Ingeniería Química, Facultad de Química, Circuito Escolar S/N, Coyoacán, Cd. Universitaria, Ciudad de México 04510, Mexico; aafdlr@ciencias.unam.mx

**Keywords:** pipitzols, isopipitzols, in silico, DFT, molecular docking, COX-2, PARP-1, cytotoxicity

## Abstract

PARP-1 and COX-2 have played important roles in several carcinomas, representing potential therapeutic targets; natural products have constituted interesting alternatives in cancer research, and complementary computational methods are relevant tools for the proposal of new molecules. Therefore, in this work, a theoretical study of a set of four derivatives of perezone and isoperezone, i.e., α-pipitzol, β-pipitzol, α-isopipitzol, and β-isopipitzol, employing quantum chemistry, bioinformatics, and docking, was performed. Conformational studies were accomplished to obtain minimum energy structures. Subsequently, they were optimized by the B3LYP hybrid method and the 6-311++G(d,p) basis set. With this same level of theory, the geometrical, electronic, and spectroscopic properties and the reactivity parameters were determined; moreover, a molecular docking evaluation was performed to determine their activity towards COX-2 and PARP-1. Additionally, a cytotoxicity activity assay was performed against various cancer cell lines; thus, α-pipitzol and β-pipitzol showed the greatest affinity for COX-2, and the α-isopipitzol exhibited two relevant interactions. Regarding α-pipitzol, it exhibited both affinity and an important interaction with PARP-1. Regarding β-pipitzol, it displayed the lowest inhibitory concentration in A549 (64.49 µM); nevertheless, α-isopipitzol presented the lowest inhibitory concentrations, 83.59 µM and 87.85 µM for U37 and MCF-7 cell lines, respectively.

## 1. Introduction

**Figure 1:**
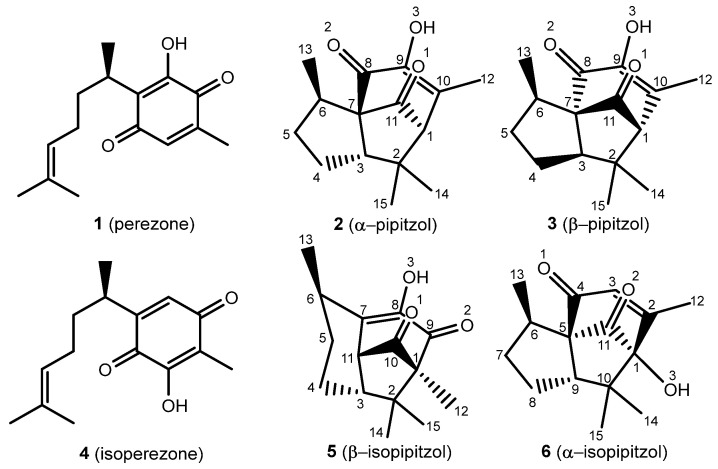
Perezone and isoperezone, and their derivatives **2**, **3**, **5**, and **6**, with assigned numeration.

Biologically active natural products have significantly contributed to medicinal chemistry since ancient times, leading to novel therapeutic strategies and chemical diversification, revealing natural products as a set of plausible drug candidates [1]. It is convenient to note the medicinal effects of the roots from *Acourtia* (previously *Perezia*) specimens and, in addition, to highlight the isolation of perezone (**1**) by Leopoldo Río de la Loza in 1852 [2]. Since then, there have been a broad variety of therapeutic applications for **1** and isoperezone, the antiproliferative, antineoplastic, and anti-inflammatory activities being the most significant [3,4]. It is important to mention the production of the following analogs: isoperezone (**4**) α-pipitzol (**2**), β-pipitzol (**3**), α-isopipitzol (**5**), and β-isopipitzol (**6**) (Figure 1)**.**

The α- and β-pipitzols are stereoisomeric secondary metabolites attained by *exo* [4 + 2] cycloadditions of **1**; these transformations are easily reproducible in the laboratory, in the presence of diverse Lewis acids [5]. Regarding **4**, it is obtained by a catalyzed 1,2-dicarbonyl transposition of **1**, and can be chemically transformed to β-isopipitzol (**6**) from an *endo* [4 + 2] cycloaddition [6]; meanwhile, the α isomer (**5**) is produced by a dipole addition [1 + 3] from perezone [7].

On the other hand, theoretical methods employing DFT calculations have consolidated their importance in chemical research, as they have been proven to achieve close similarities with experimental data, due to robustness, greater accuracy, and more efficient approximations [8,9]; in addition to the convenient acquisition of geometrical properties, electronic structures, and spectroscopic properties [10,11]. Moreover, physicochemical properties, through the Lipinski rule of five, biological potential prediction, and molecular docking analysis, are appropriate key tools in medicinal chemistry for the discovery of novel molecular targets with potential therapeutic applications [12,13,14,15].

Taking as background the described activities of **1** and **2** [2,3,4], and to our knowledge after a profound literature search, the almost absent pharmacological and theoretical studies related to pipitzols and isopipitzols, the goal of this work is to perform the following: first, an in vitro cytotoxicity assay with five different human tumor cell lines to evaluate the compound’s potential growth inhibition effect; and second, a computational study, carrying out a complete in silico study, analyzing the geometrical evaluation and characterization of the target molecules (**2**, **3**, **5**, **6**) employing DFT-methodology at the B3LYP/6-311++G(d,p) [3,4,9] level, as well as studying their predicted physicochemical and biological properties and their possible affinity to the active sites of the enzymes PARP-1 and COX-2, through a molecular docking analysis [16,17], highlighting the relation between inflammation and cancer [18,19].

## 2. Results and Discussion

### 2.1. Optimization of the Target Molecules ***2***, ***3***, ***5***, and ***6***

**Figure 2:**
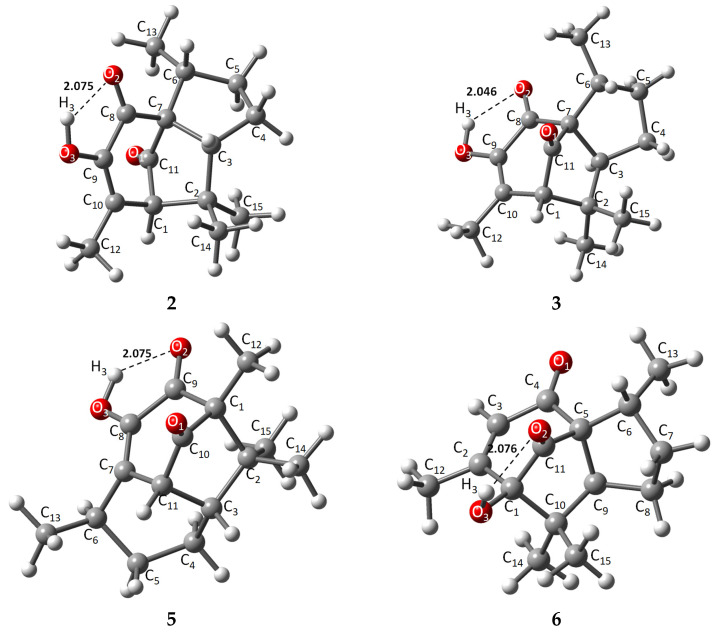
Theoretically optimized representations for **2**, **3**, and **5**, **6** most stable conformers.

The energies of the optimized structures and the geometrical representations (**2**–**3**, **5**–**6**) are summarized in Table 1 and Figure 2, respectively. When comparing energies between α and β pipitzol isomers, **2** (−507,990.37 kcal/mol) showed the most stable energy value, followed by **3** (−507,989.47 kcal/mol), while **5** (−507,975.32 kcal/mol) exhibited the most stable energy value from **5** and **6** (−507,975.16 kcal/mol) isoperezone isomers. **2** and **3** displayed similar stability, with a difference of 0.1033 kcal/mol, these stereoisomers being the most stable compounds. It should be noted that these conformers depicted strong (1.5–2.2 Å) intramolecular hydrogen bonds [20] (O2-H3, 2.075, 2.076, and 2.046 Å, respectively), as well as a greater proximity between the C13 and O2 in **2** and **3** (α and β pipitzols).

### 2.2. Molecular Geometry for ***2***, ***3***, ***5***, and ***6***

#### 2.2.1. Bond Length (Å)

Geometrical parameters were calculated using B3LYP/6-311++G(d,p) for the optimized **2**, **3**, and **5**, **6** molecules. Additionally, experimental bond lengths for every compound are denoted in Table S1, Supplementary Materials, considering previously reported single-crystal X-ray analysis [7,21,22] to relate the theoretical data accuracy. The atom numbering is indicated in Figure 1.

X-ray diffraction information was used to compare the theoretical method employed with experimental data. The main structure of all compounds is represented by a tricyclic with two five- or six-membered *cis*-fused rings and a five- or six-membered ring with its corresponding carbonyl bridge (C1-C11). Diastereoisomers **2** and **3** showed very similar theoretical bond length data, where distances from representative carbonyl bridges C7-C11 and C1-C11 were 1.545, 1.539, and 1.526, 1.523 Å, respectively; these distances were longer compared to experimental 1.524, 1.557, and 1.511, 1.491 Å, except for 1.557 Å for **3**. Similarly, the same bridges for **5** (C1-C10, C10-C11) and **6** (C1-C11, C11-C5) are 1.527, 1.510, and 1.526, 1.521 Å, respectively, where most similarities were found compared to experimental data, highlighting the fact that **2** has the longest bridge distances due to a slightly different spatial distribution at the principal ring. Carbonyl bonds located in the seven-membered ring showed bond lengths of around 1.2 Å [23], consistent with general carbon–oxygen double bonds, while a C-O3 single bond is found between 1.357 and 1.412 Å, in which **6** exhibited the longest length, taking part in intramolecular hydrogen bond formation with O2, increasing the molecule’s stability by chain formation along their axis [7,21,22].

Intramolecular hydrogen bonds O2-H3 exhibited similar distances, where **3** showed the strongest intramolecular length as an approach from the oxygen atom towards H3 (2.046 Å), compared with their stereoisomer **2** (2.075 Å) and the isoperezone derivatives **5** and **6** (2.075 and 2.077 Å), with the hydroxyl group as donor and the carbonyl as acceptor [20].

#### 2.2.2. Bond Angles (°)

Calculated bond angles for all compounds were obtained. The corresponding results are listed in Table S2, Supplementary Materials, showing the most important bond angles for each compound.

In addition, calculated bond angles for perezone and isoperezone derivatives were obtained. The corresponding results show that theoretical angle values agreed with the experimental X-ray diffraction values, where small deviations were found at the principal tricyclic ring in **2** and **3**, in which the C3-C4-C5 angle increased slightly (103.9° and 106.2°), respectively, observing the same behavior for C7-C8-C9 and C7-C8-C9 moieties. The atoms involved in the carbonyl bridge and the ring fusion, mainly C1-C11-C7 in **2** and **3**, C1-C10-C11 in **5**, and C1-C11-C5 in **6**, indicated similar theorical angles within α derivatives **2** and **5** (103.7° and 104.8°, respectively), while in β derivative **6**, a decrease was observed (99.8°), comparable to experimental information [7,21,22]. **6** was the only derivative where the intramolecular hydrogen bond formation was located between O2-H3 in the carbonyl bridge, having the possibility to narrowly modify the ring’s angles. Similarly to the previously mentioned angle, theoretically, O1-C11-C7 (**2**, **3**), O1-C10-C1 in **5**, and O2-C11-C5 for **6**, an increase in the corresponding angle in **6** was noted (130.0°), in contrast to 128.8°, 125.5°, and 127.4° from the rest of the derivatives. Unfortunately, no experimental information on O2-C11-C5 in **6** is reported in the literature; nevertheless, a good approach of the theoretically obtained data for **2**, **3**, and **5** at the same angle with experimental data might indicate significant accuracy.

In general, similar bond angles were identified among α and β diastereomers (**2**, **3**). In addition, C3-C7-C11 showed an appreciable increase in **3**, exhibiting specific differences in the tricyclic ring observed in experimental information (114.1°, 120.0°), including C7 and C11 as enantiomeric atoms. Both diastereomers’ five-membered rings contain the methyl C13 that has been described as a half-chair conformation to reduce intramolecular steric repulsions with neighboring oxygen atoms O2 [21]. **5** has a similar half-chair conformation as **2** and **3**, where resemblance to theoretical data was detected, mostly comparing the reported X-ray information from the principal structure C5-C6-C7 (105.6°/105.7°), C7-C8-C9 (121.1°/121.0°), C8-C9-C1 (117.1°/117.8°), and, where oxygen atoms are involved, O1-C10-C1 (126.3°/127.4°) and O3-C8-C7 (121.7°/123.2°).

Similarly, **6** is represented by the comparison with representative angles from the tricyclic framework, achieving consistent accuracy with theoretical data, representing a good geometrical description at the proposed quantum mechanical level. C11-C5-C9 represents the implicated *cis*-fused atoms C5-C9 with angles of 101.8°/100.6°. Characteristic oxygen-containing angles are not described in the literature. Additionally, carbonyl carbon–oxygen angles O1-C4-C5, O1-C4-C3, O2-C11-C1, and O2-C11-C5 resulted in values between 121° and 124°, with O2-C11-C5 as an exception, in which the angle was deviated to 130.0°, as a possible result of a distorted envelope conformation within the other rings [7].

#### 2.2.3. Dihedrals (°)

Dihedral angles of pipitzols and isopipitzols are shown in Table S3, Supplementary Materials Section. It is worth mentioning that experimental data for **5** and **6** are not reported. A dihedral angle is usually referred to as the angle between two planes that pass through the same bond. Dihedrals describe the angles around double bonds, the preferred angles being between 0° and 180°. On the other hand, there is less preference at specific angles for single bonds and rotation [24].

Most relevant reported dihedrals are in the tricyclic ring for **2** and **3**, where close values were shown for clockwise rotation dihedrals in C3-C4-C5-C6 and C5-C6-C7-C3 bonds. On the other hand, negative counterclockwise rotations were reported for C4-C5-C6-C7 and C7-C3-C4-C5 dihedrals. In general, selected dihedrals where double bond atoms C8 and C9 are considered, such as C8-C9-C10-C1 (1.0°) and O2-C8-C9-O3 (−1.7°), suggesting a dihedral closer to 0° and 180°—as mentioned above, preferred angles. Similarly, for compound **6**, dihedrals among C2 and C3 are closer to planarity, while for compound **5**, bonds containing C7 and C8 atoms, such as C5-C6-C7-C8 (120.9°) and C6-C7-C8-C9 (−154.1°), are distant from planarity, due to structural differences in β-isomer **5**.

### 2.3. Electronic Parameters for ***2***, ***3***, ***5***, and ***6***

#### 2.3.1. Natural Atomic Charges of the Target Compounds

Atomic charges for all compounds were evaluated by the Natural Population Analysis (NPA) scheme, as shown in Table S4, Supplementary Materials. Results have indicated that the most negative charge is localized on the oxygen atom of the hydroxyl group O3 in compounds **2**, **3** (−0.681 e^−^) and **6** (−0.768 e^−^), explained by a strong intramolecular hydrogen bond within the hydroxyl group and the adjacent carbonyl group (OH⋅⋅⋅O=C) in target molecules. On the other hand, oxygen atom O1 was the most negative atom in compound **5** (−0.681 e^−^). The same behavior was observed in the electrostatic potential map, as the sites with the highest electronic density are found around oxygen atoms in all compounds.

**Figure 3:**
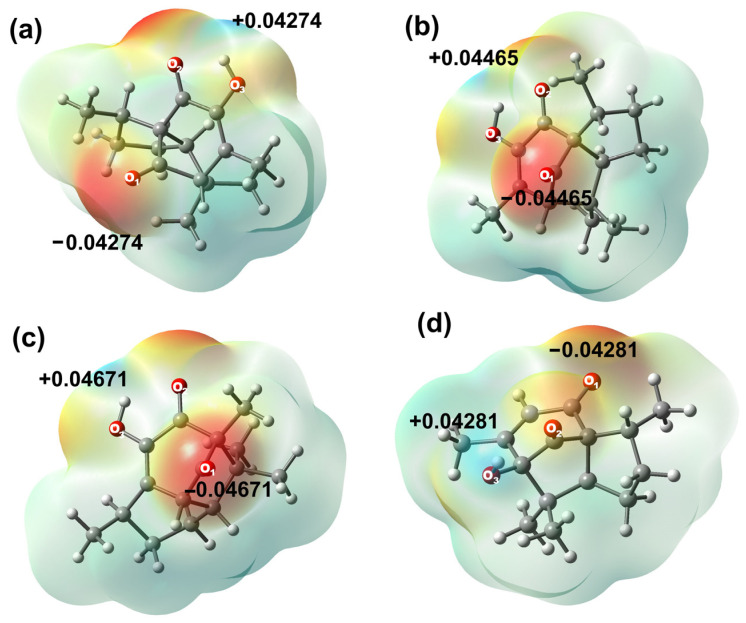
Molecular electrostatic potential map in (eV) of (**a**) **2**, (**b**) **3**, (**c**) **5**, and (**d**) **6**.

It is worth noting that atoms C12 to C15 in all compounds exhibited natural atomic charges from −0.571 e^−^ to −0.603 e^−^, C12 being the most polarized carbon in both molecules, explained by the electronic delocalization towards the unsaturated α-β system. On the other hand, sites with a high positive charge are localized at the carbon atoms of the carbonyl group (C11 for **2**, **3**, **6**, and C10 in **5**), due to the inductive effect exerted by the oxygen atom. C11 from compounds **2** and **3** ($\bar{x}$ = 0.6435 e^−^), **6** (0.591 e^−^), and C10 from **5** (0.631 e^−^) exhibited similar behavior. C11 from **6** was the least positively charged carbon among all derivatives, owing to the intramolecular hydrogen bond interaction between OH-H3, which is only observed at the carbonyl bridge in derivative **6**, Figure 3.

As a complement, the second atoms in order of positive charge correspond to carbonyl carbon C8 for **2** and **3** ($\bar{x}$ = 0.5325 e^−^), C9 for derivative **5** (0.529 e^−^, by intramolecular hydrogen bond formation), and C4 for derivative **6** (0.397 e^−^). Molecular electrostatic potential (MEP) has suggested that hydroxyl group hydrogens are the most positively charged in all the studied molecules. Diastereomers **2** and **3** have indicated great similarity, resulting in brief variations according to their natural atomic charges. On their behalf, **5** and **6** cannot be fully compared due to their structural differences; however, carbonyl carbons, oxygen atoms, and methyl substituents are atoms with similar charge values.

#### 2.3.2. HOMO-LUMO Orbitals Distribution

The highest occupied molecular orbital (HOMO) and the lowest unoccupied molecular orbital (LUMO) were used to determine the chemical stability of the target molecules, as described in Table 2. Molecular orbital and GAP energy were calculated to describe reactivity and biological activity in chemical systems. Compound **5** was the most stable compound of all derivatives, with a GAP value of 5.17 eV, while compound **6** was the most reactive (4.59 eV), showing the following reactivity order: **5** < **2** < **3** < **6**. Among α diastereomers, **2** was the most stable by 0.09 eV. Moreover, **3** exhibited greater stability than **5** for β compounds by 0.47 eV.

**Figure 4:**
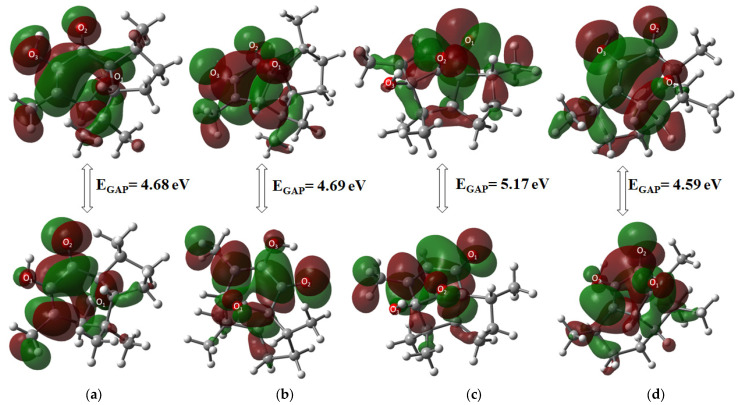
Molecular orbital HOMO-LUMO distribution of (**a**) **2**, (**b**) **3**, (**c**) **5**, and (**d**) **6**.

General molecular orbital distribution is found in the six-membered ring for the HOMO orbital, located upon oxygen atoms as an electronic donor region, whereas the LUMO orbital acceptor region in all compounds is distributed all over the ring structure (Figure 4).

### 2.4. Chemical Parameters

Table 3 shows the neutral, positive, and negative charges calculated by DFT methodologies, from which reactivity properties were obtained. Assorted physicochemical properties are employed in the literature to describe molecules’ chemical behavior, including two theoretical descriptors based on charges, ionization energy, and electron affinity of atoms [25], hardness (η), and electrophilicity index (ω). These last two parameters possess adequate information structure, stability, and compound reactivity [26]. According to the literature, a large GAP energy value corresponds to a hard molecule, being related to molecular orbital information [25]; η values increase in the order **3** (3.79) < **6** (3.98) < **2** (4.05) < **5** (4.34), which is consistent with the maximum hardness principle (MHP), which states that the most stable conformer with minimum energy is the hardest [27,28], **5** being the hardest in this case.

According to a smaller band gap energy and the small hardness data of the molecules, **2** and **6** could show chemical and possibly biological activity, since they can be reactive to change [29].

It is of great interest to note that the electrophilicity index increases in the order **5** (2.17) = **6** (2.17) < **2** (2.23) < **3** (2.41), where **2** and **3** diastereomers were being more electrophilic than **5** and **6** isoperezone derivatives. Additionally, the electronic chemical potential (μ) is associated with the feasibility of a system to exchange electron density with the environment at the ground state, implicating a large μ with a good electronic acceptor and a small μ with a good electronic donor [30]. Therefore, electronic chemical potential increases in the order **2** (−4.25) < **3** (−4.27) < **5** (−4.34) < **6** (−4.40). **2** and **3** exhibited the lowest chemical potential, while μ increased in **5** and **6** derivatives. In that way, a good electrophile, such as **6**, has a high value of μ (−4.40) and a low η value (3.98).

Regarding ionization potential (IP), all target compounds showed positive values. The resulting values are located between 8.06 and 8.68 eV, increasing in the order **3** (8.06) < **2** (8.29) < **6** (8.38) < **5** (8.68), showing that, once more, **2** and **3** perezone derivatives are more susceptible to losing electrons [31]. On the other hand, electronic affinity (EA) was obtained as the difference between the neutral energy and the negative energy species of the optimized structure. EA values were localized in the order **5** (−0.01) < **2** (0.20) < **6** (0.42) < **3** (0.48); only EA from derivative **5** was a negative value.

Finally, electronegativity (χ) is considered an empirical and very useful concept in the context of chemical reactive behavior that quantifies the ability of an atom to attract electrons [32], being described by Mulliken as 0.5 (IP + EA) [33]. The electronegativity range was identified between 2.2451 and 4.4030, meaning that **6** is the compound with the highest tendency to attract electrons towards itself.

### 2.5. Spectroscopic Properties for Target Compounds ***2***, ***3***, ***5***, and ***6***

Theoretical approximations related to spectroscopic properties were attained for the most stable conformer of each molecule in the gas phase. Vibrational modes in infrared spectrophotometry are presented, as well as chemical shift values in nuclear magnetic resonance, considering a comparative study between the calculated theoretical and experimental values. According to the literature search, there are no previous reports about theoretical IR vibrational frequencies, NMR chemical shifts, and their correlation with experimental results for **2**, **3**, **5**, and **6** derivatives.

#### 2.5.1. Infrared Spectrophotometry

**Figure 5:**
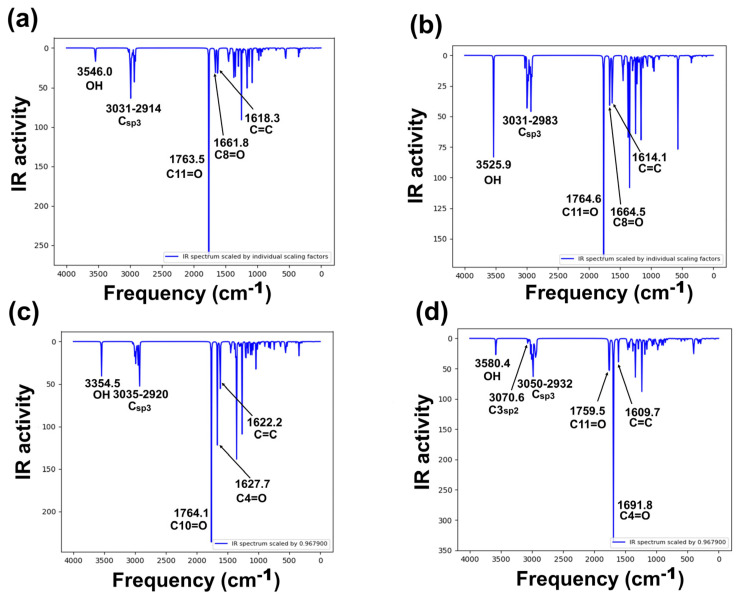
Calculated vibration spectra of studied derivatives, computed at B3LYP/6-311++G(d,p) level of theory: (**a**) **2**, (**b**) **3**, (**c**) **5**, and (**d**) **6** [35].

Target compounds have 36 atoms and 108 vibrational modes, where all vibrations were active in infrared absorption. The most important infrared theoretical and experimental wave numbers [5,34] are summarized in Figure 5a–d, Table S5, Supplementary Materials Section. The analysis focuses on specific vibrational modes: cyclopentanone, six-member enolized α-diketone, and O-H bands for all molecules. Even though differences in stereochemistry between diastereomers **2** and **3** are presented, the reported vibrational modes in the literature were similar. Harmonic frequencies, with a scaling factor (0.9679) of the selected modes, were compared with the available experimental information.

Vibrational frequency deviation percentage from all compounds resulted in good theoretical–experimental correlation, the O-H band corresponding to the frequency with the greatest differences, although in all cases, deviation was lower than 3%. Theoretical frequencies (Figure 5) showed in all compounds a band at approximately 3500 cm^−1^, assigned to the O-H group. These values are consistent with previously reported experimental frequencies in the range 3450–3500 cm^−1^ [5,34]. Cyclopentanone assignment was theoretically identified at 1764–1765 cm^−1^, corresponding to the carbonyl assignment zone, having agreement with those previously reported, at approximately 1756–1760 cm^−1^. Regarding the six-member enolized α-diketone, two vibrations were detected in the range 1609–1691 cm^−1^, where the theoretical frequencies of this band in compound **6** were higher than those of compounds **2**, **3**, and **5**. The results demonstrated a good theoretical–experimental correlation for compounds **2**, **3**, and **5**. However, as mentioned above, for **6**, no information related to infrared spectra has been reported yet. In this sense, a complete spectroscopical characterization is in progress in our research group.

#### 2.5.2. Nuclear Magnetic Resonance ^1^H and ^13^C

**Figure 6:**
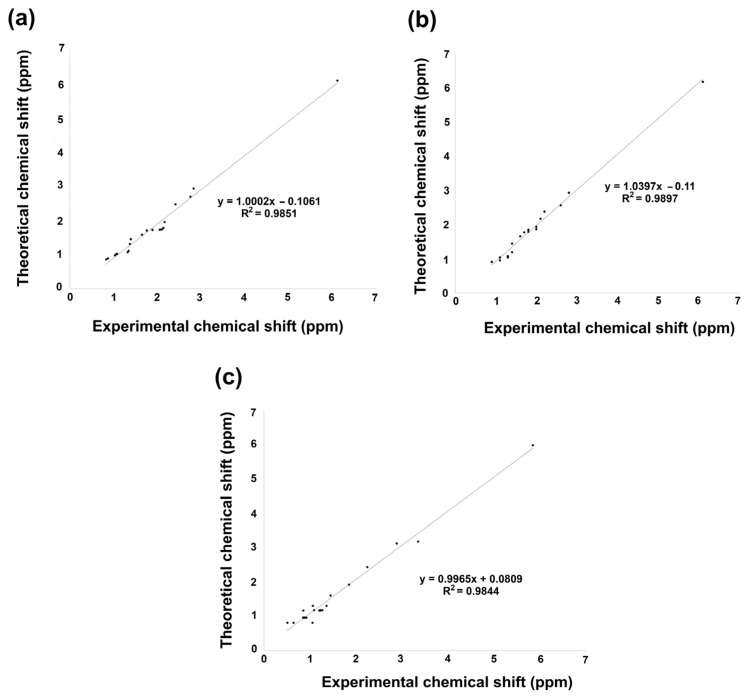
^1^H NMR linear regression between theoretical calculation by B3LYP/6 311++G(d,p) and experimental data for (**a**) **2**, (**b**) **3**, and (**c**) **5**.

**Figure 7:**
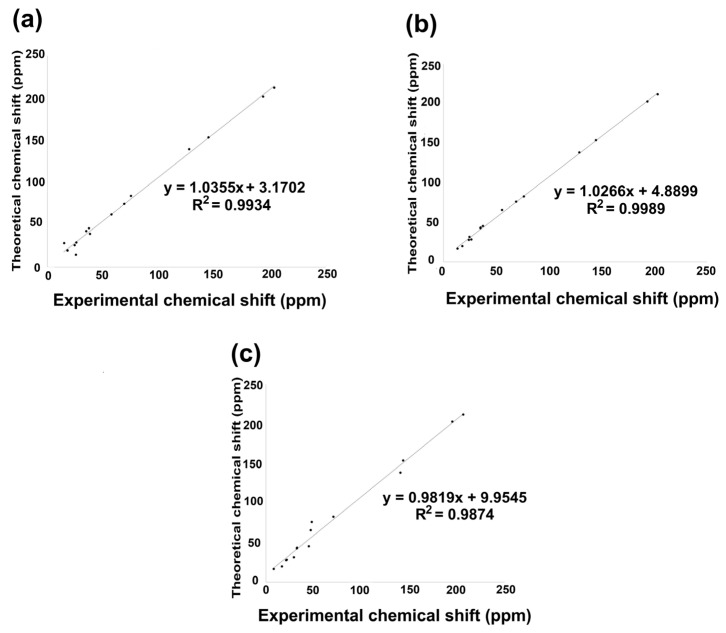
^13^C NMR linear regression between theoretical calculation by B3LYP/6 311++G(d,p) and experimental data for (**a**) compound **2**; (**b**) compound **3**; (**c**) compound **5**.

Nuclear magnetic resonance (NMR) theoretical chemical shifts for ^1^H and ^13^C in the gas phase of the studied derivatives were also obtained. Theoretical values were calculated using the B3LYP functional with 6–311++G(d,p) basis set and the GIAO method. Figure 6 and Figure 7, Tables S6 and S7 in the Supplementary Materials Section, display the linear regression correlation between theoretical and experimental shifts for **2** and **3** for a comparative analysis [36]. It is worth noting that ^1^H NMR theoretical results for compounds **2**, **3**, and **5** exhibited excellent correlation with the experimental data. On the other hand, compound **6** could not be correlated, as experimental information is not reported; consequently, its spectroscopical characterization is in progress in our research group.

The linear regression of ^1^H NMR information is shown in Figure 6a–c. Hence, according to the analysis, ^1^H chemical shift suggested for **2** a regression coefficient of 0.9851, a standard deviation of 1.1491, and a typical error of 0.1453 ppm. On the other hand, **3** exhibited a slightly better regression coefficient of 0.9897, a standard deviation of 1.1319, and a typical error of 0.1174 ppm. Finally, **5** achieved a regression coefficient of 0.9844, a standard deviation of 1.2184, and a typical error of 0.1581 ppm. The equations to describe the fit are *δ*_theoretical_ = 1.0002*δ*_calculated_ − 0.1061 ppm, *δ*_theoretical_ = 1.039*δ*_calculated_ − 0.11 ppm, and *δ*_theoretical_ = 0.9965*δ*_calculated_ + 0.809 ppm, respectively, for **2**, **3**, and **5**.

The linear regression analysis of the ^13^C NMR data set is shown in Figure 7a–c. Consequently, the ^13^C chemical shift indicated an excellent regression coefficient of 0.9934, with a standard deviation of 64.072 and a typical error of 5.3958 ppm for **2**. Nevertheless, **3** showed a regression coefficient of 0.9989, a standard deviation of 64.030, and a typical error of 2.207 ppm, while **5** exhibited a regression coefficient of 0.9874, a standard deviation of 65.326, and a typical error of 7.7603 ppm. The equations that describe the fit are *δ*_theoretical_ = 1.035*δ*_calculated_ + 3.1702 ppm, *δ*_theoretical_ = 1.0266*δ*_calculated_ + 4.889 ppm, and *δ*_theoretical_ = 0.9874*δ*_calculated_ + 9.9545 ppm, respectively, for **2**, **3**, and **5**. The lowest field nuclei are carbonyl carbons by oxygen deprotection phenomena, followed by carbonyl adjacent carbons [32].

### 2.6. Biological Activity Prediction of ***2***, ***3***, ***5***, and ***6***

#### 2.6.1. Physicochemical Lipinski Rule of Five and ADME-Tox Properties, and PASS Biological Target Prediction

Prediction of physicochemical and pharmacokinetic properties was obtained and evaluated regarding the Lipinski rule of five, Table 4, a highly employed method to estimate solubility, membrane permeability, and oral absorption in drug development [37].

Table 4 displays the most important physicochemical properties included in the Lipinski rule of five, where all target compounds exhibited no violations to the “drugability” guideline, considering the rule accomplishment as a higher permeation probability through passive transport [40]. The molecular weight from the four compounds, as there are isomers among them, indicated a lower value than 500 Da, as smaller molecules are preferred to facilitate barrier crossing. The calculated logP changed from one isomer pair to another, resulting in a logP value of 2.23 for **2** and **3**, and 2.16 for **5** and **6**, these values being lower than those of **5**, as hydrophilic–hydrophobic balanced distribution among aqueous and lipophilic media is important in the cellular environment [40].

H-bond donors and acceptors (nON and nOHNH) are set in the Lipinski rule of five as no more than five H-bond donors and ten H-bond acceptors; thus, each molecule has an H-donor and three H-acceptor sites [40]. Finally, topological polar surface area was evaluated as an additional physicochemical property, representing the sum of polar atom surfaces in a molecule, being employed as another property to analyze drug transport and bioavailability [41]. Supplementary chemo-informatics filters such as Veber and Egan filters have indicated values under 140 and 131.6 Å^2^ as a characteristic of a well-absorbed compound in humans [42] and consequently, a good bioavailability. The four target compounds had a topological polar surface area (TPSA) value under 140 Å^2^.

Pharmacokinetic property prediction employing SwissADME and Osiris Property Explorer [38,43] is presented in Table S9, Supplementary Materials. Predictive tools suggested that gastrointestinal absorption is favored, showing advantages with possible oral administration. Nevertheless, all compounds indicated blood–brain barrier permeation, in which compounds **5** and **6** are P-glycoprotein (P-gp) substrates. Experimental evaluation must be conducted to evaluate the biological effects of the brain barrier permeation, especially with compounds **2** and **3**, where P-gp does not have an extrusion effect. Regarding biotransformation and the CYP450 enzymatic system, **2** and **3** exhibited inhibition activity for the CYP2C9 isoform, where attention should be paid, as CYP2C9 is the major enzyme responsible for the metabolic clearance of several drugs with a narrow therapeutic index [44].

Finally, toxicity prediction was done, resulting in no mutagenic, tumorigenic, irritant, or reproductive effects with any of the studied compounds. A drug score is additionally obtained by Osiris Property Explorer, based on the combination of three physicochemical (logP, logS, and molecular weight) and toxicity property predictions in a grand total, seeking to achieve the closest score to 1 [43]. Compounds **2** and **3** achieved a slightly better score of 0.583 than **5**, **6** of 0.547.

#### 2.6.2. Biological Activity Prediction

Biological target prediction was established by the usage of database empirical predictors, Table S10a,b, Supplementary Materials. Pass Online [13,45,46] was the main tool for potential biological activity, providing a closer approach to therapeutic targets, preliminary to primary biological testing. Pass Online results are indicated as probabilities, where Pa refers to the probability “to be active” and Pi defines the probability “to be inactive”. In this context, a Pa value higher than 0.7 described a higher probability of showing activity.

According to the Pass Online prediction, **2** and **3** exhibited similar potential activity as polarization agents, involving macrophage activation and exerting antitumor and inflammatory responses [47] (Pa = 0.844, Pi = 0.001), and as possible anti-neoplastic agents (Pa = 0.844, Pi = 0.001), having recent advances and currently used treatments of this kind of drugs derived from natural products [48,49]. The aim of studying both PARP-1 and COX-2 proteins was to evaluate potential effects in different enzymes expressed in multiple cancer cells [50].

#### 2.6.3. Molecular Docking Study

**Figure 8:**
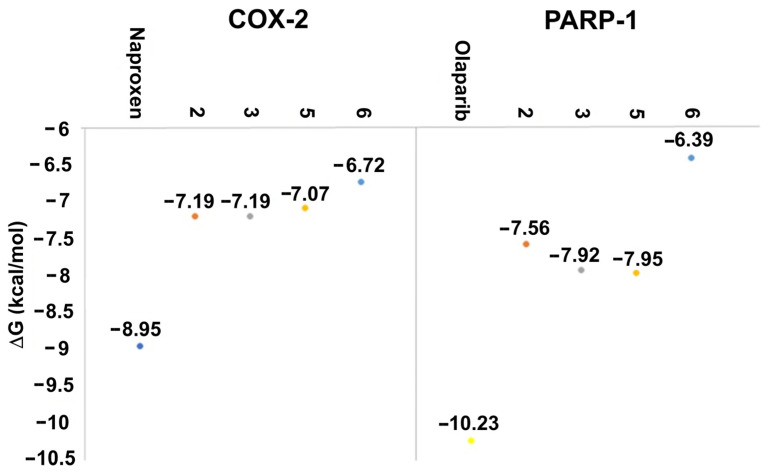
Docking results of the target compounds with COX-2 (PDB ID: 3NT1) and PARP-1 (PDB ID: 1UK0). ΔG values obtained for naproxen (blue), olaparib (yellow), **2** (red), **3** (grey), and **5** (orange), **6** (light blue).

The affinity and binding modes of target compounds with human COX-2 and PARP-1 were verified by the molecular docking analysis. Consequently, to accomplish a comparative study with known co-crystallized ligands, docking studies were carried out with naproxen and olaparib as reference compounds, showing ΔG results in Figure 8 and Table S8, Supplementary Materials.

The COX-2 active site was identified by the pocket amino acid residues. The cyclooxygenase-2 (COX-2) isoform is responsible for prostaglandin production, employing selective inhibitors to control and decrease inflammation processes. The target hydrophobic pocket is in the active site cavity from Arg120 to Tyr385, having a larger binding cavity covering the amino acids Val523, Val434, Leu503, and Arg513 at a hydrophilic portion, allowing interactions with polar and non-polar moieties and greater selectivity compared to cyclooxygenase-1 isoform [51].

**Figure 9:**
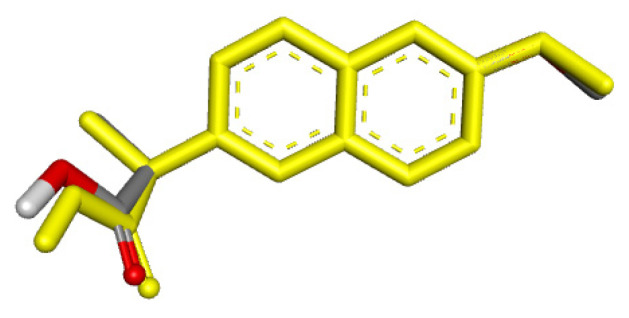
RMSD for co-crystallized ligand naproxen (yellow) concerning reference ligand at the crystal structures (gray) for illustrating a good docking solution (RMSD ≤ 2.0 Å) as COX-2 docking validation, also the oxygen atoms are indicated in red color.

Preliminary to the molecular docking assay, validation of the docking protocol was required through the re-docking of the co-crystallized ligand in the original protein structure, obtaining an RMSD value of 0.2910 Å (Figure 9). Root-mean-square deviation (RMSD) was employed for the evaluation of the differences between the obtained re-docking and the co-crystallized pose of the same ligand molecule. RMSD values < 2.0 Å correspond to a good docking solution [52].

**Figure 10:**
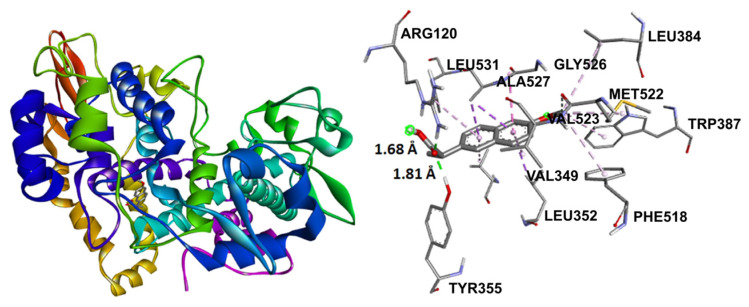
Docking binding mode and main interactions with human COX-2 of naproxen.

**Figure 11:**
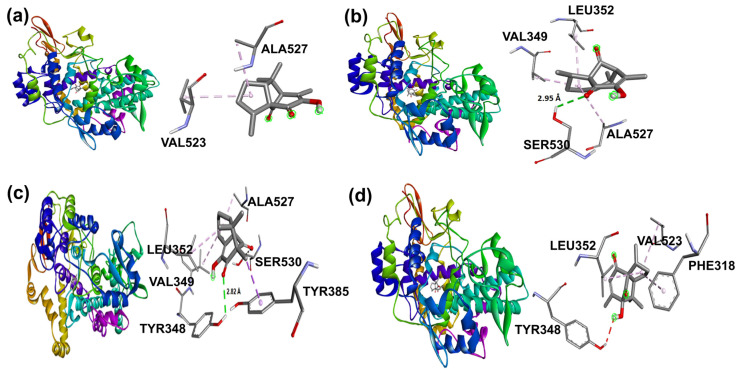
Docking binding modes and main interactions with human COX-2 of (**a**) **2**, (**b**) **3**, (**c**) **5**, and (**d**) **6**.

Naproxen molecular recoupling resulted in the amino acid interactions shown in Table S8, Supplementary Materials, where coincidence was found with previously reported amino acid residues in the pocket cavity [53,54]. The COX-2 evaluation with naproxen represented several important interactions, such as hydrogen bonds with Tyr355 and Arg120, and mostly hydrophobic interactions with Leu531, Trp387, Val349, and Leu352, where Val523 denoted a selective interaction with the COX-2 isoform, Figure 10. On the other hand, target compounds resulted in lower stability interaction energies than naproxen (−8.75 kcal/mol), showing mainly hydrophobic interactions with pocket residues. It is worth mentioning that selective interaction with Val523 is present in **2** and **6**; both compounds corresponded to α-isomers. **2** and **3** exhibited the greatest stability energy values (−7.19 kcal/mol), while **5** and **6** showed a ΔG increase (−7.07, −6.72 kcal/mol), consequently reducing their interaction stability (Figure 11).

In addition, β-isomers have revealed similar hydrophobic interactions, including Ser530 (for **3** and **5**) and Tyr385 (for **5**) [51] amino acid residues as part of the enzyme hydrophobic cavity. These results indicated that there were differences in protein–ligand interactions in view of the studied isomer and its binding mode in the cavity site.

**Figure 12:**
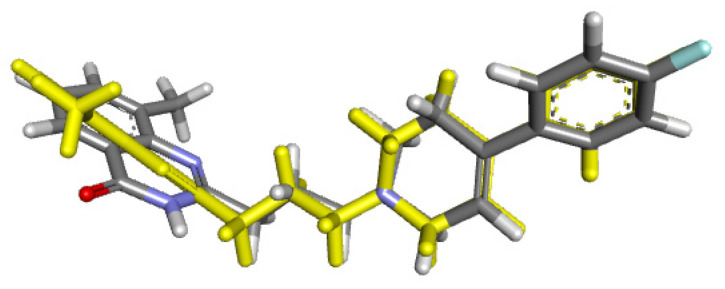
RMSD for co-crystallized ligand FR257517 (yellow) concerning reference ligand at the crystal structures (gray) for illustrating a good docking solution (RMSD ≤ 2.0 Å) as PARP-1 docking validation, also the oxygen, nitrogen, and fluor atoms are indicated in red, blue, and light blue colors.

The second protein studied was PARP-1, an enzyme responsible for cellular processes and metabolism regulation, acting mainly towards DNA damage responses and chromatin remodeling [55]. The PARP family, **1**–**6**, in mammals, share a conserved His-Tyr-Glu at their catalytic cavity located at the nicotinamide-binding pocket. Before the molecular coupling assay, validation of the docking protocol was performed through the re-docking of the co-crystallized ligand FR257517 from the original 3D structure employing the same procedure for the docked compounds, (Figure 12; RMSD 1.1153 Å).

**Figure 13:**
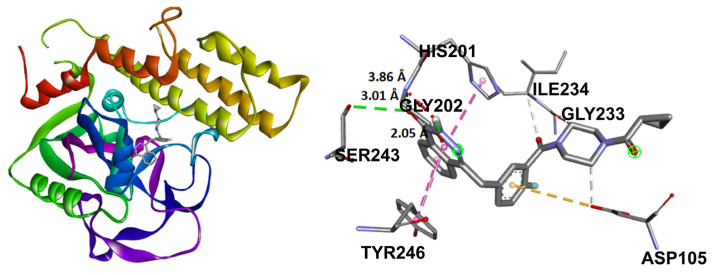
Docking binding mode and main interactions between PARP-1 and olaparib.

Olaparib, the reference compound, has shown affinity to the PARP-1 crystal protein with an interaction energy of −10.23 kcal/mol, displaying a hydrogen bond with Tyr235 and π-π interactions with His201 (Figure 13).

**Figure 14:**
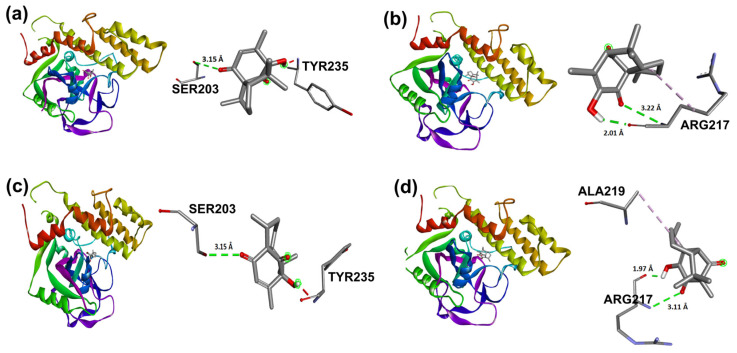
Docking binding modes and main interactions with human PARP-1 of (**a**) 2; (**b**) 3; (**c**) 5; (**d**) 6.

Target compounds resulted in binding modes with lower stability binding energies, diminishing amino acid residue interactions. Compound **2** displayed the second greatest stability of all compounds (−7.56 kcal/mol) and a hydrophobic interaction with Tyr235, where the α isomer presented a favorable binding conformation at the triad site. Nevertheless, **3**, **5**, and **6** did not demonstrate important interactions with the catalytic pocket, showing minor affinity to amino acid residues at the catalytic triad (Figure 14).

### 2.7. In Vitro Cytotoxicity of ***2***, ***3***, ***5***, and ***6***

**Figure 15:**
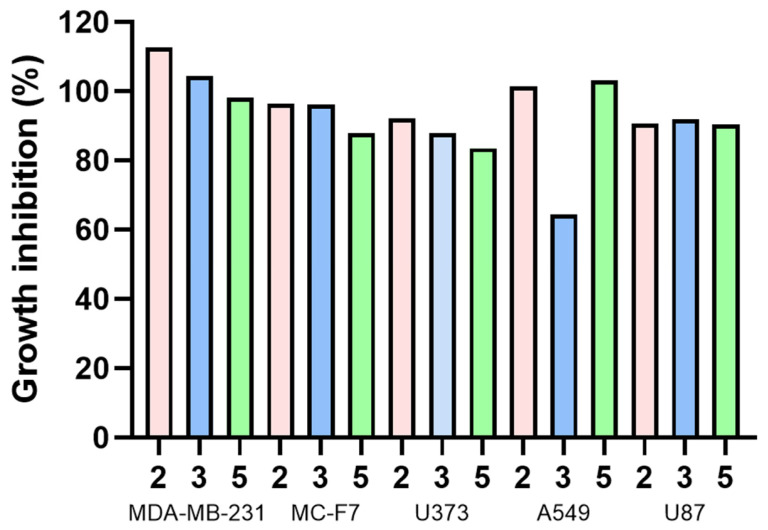
Growth inhibition (%) of different human tumor cell lines for compounds **2**, **3**, and **5**.

Compounds **2**, **3**, and **5** were tested for cytotoxic activity (Table 5, Figure 15) in five tumor cell lines: MDA MB 231, MCF-7, U373, A549, and U87, where tumor cell growth inhibition was expected.

The cell viability percentages obtained indicate that these compounds did not cause mortality at 25 μM in the cancer cells used. This concentration has been suggested as significant to reaching further analysis [56]; therefore, derivatives **2**, **3**, and **5** were not considered candidates for future studies. However, **4** displays the lowest inhibitory concentration of all compounds for A549 (64.49 µM), while **5** achieved the lowest inhibitory concentration in the MCF-7 cell line, reaching 83.59 µM. According to previously reported information for olaparib (positive reference), its inhibitory concentrations were described in several sources: 6.9 ± 1.1 and 5.8 ± 1.6 µM concentration with 41.9 ± 9.7% and 43.3 ± 3.8% inhibition at MDA-MB-231 and MCF-7 cell lines, respectively [57]; 11.92 µM at A549 tumor cell lines [58]; and 27.7 ± 1.3 µM with U87 tumor cells lines [59], through MTT assay. U373 cell line assays are not reported with olaparib. Olaparib is still more potent than **2**, **3**, and **5**.

On the other hand, closer analogs to compounds **2**, **3**, and **5** studied with similar tumor cell lines were **1** and **4**. Structural differences among them are evident; the analysis pointed out that the tricyclic *cis*-fused rings at the quinone portion from all compounds contribute to the main interactions with COX-2 and PARP-1, being justified by the intramolecular addition of the side-chain double bond of **1** and the catalyzed intramolecular cycloaddition of **4**, that considerably decreased the available binding sites with the active pocket from the target proteins (Figure 12), observed since the molecular coupling analysis. Additionally, diastereomers **2** and **3** did not show greater ΔG differences between them; however, the in vitro assay exhibited significant growth inhibition variation, mainly at A549 cell lines, where spatial and structural distribution displayed a relevant attribute. **1** has been studied in similar cancer cell lines, exerting inhibitory concentrations of 143.8, 7.8, and 53.2 µM in A549, MDA-MB-231, and MCF-7 cell lines, respectively [9]. An additional assay had suggested an IC50 value of 13.2 µM for **1** when using MDA-MB-231 cultured cells [60]. It is worth noting that inhibitory concentrations at A549 cell lines for compounds **2**, **3**, and **5** are lower than the reported concentration for **1**; however, for the rest of the cell lines, IC50 values are higher than those for **1**. Recently, **4** was evaluated against MDA-MB-231 cells, having achieved 19.31 ± 0.5 µM as IC50 value [61]. When compared to **4**, the studied derivatives did not reach lower IC50 values.

Molecular docking offered the possibility to explain the correlation between the computational prediction and the in vitro assays, contrasting the results and highlighting the differences between **1** and **4** precursors. **1** and **4** were already described employing docking studies, resulting in ΔG values of −7.2 and −6.8 kcal/mol, respectively, for human COX-2 [4], when compared with −7.5 kcal/mol ΔG value for naproxen in the same study. According to the docking study, compounds **2**, **3**, **5**, and **6** exhibited similar energy values to **1** and **4** with the same protein crystal, suggesting a possible similar anti-inflammatory activity prediction, despite structural differences.

Nevertheless, foregoing research for compound **1**, related to PARP-1 employing molecular coupling studies, showed a −7.24 kcal/mol ΔG value, having established hydrogen bond interaction with the lateral chain of Tyr235, Glu327, and Tyr246 and hydrophobic interactions with Ala237 [3]. In this case, intramolecular cycloaddition resulted in the loss of binding sites in compounds **2** and **3**, only showing a Tyr235 interaction for **2**, which also explained the increased inhibitory concentrations for compounds **2** and **3** in contrast to **1**.

Thus, **4** has not been studied with PARP-1; however, its isopipitzol analogs did not show great affinity or relevant interactions to PARP-1, justifying the in vitro results with cancer cell lines.

## 3. Materials and Methods

### 3.1. Quantum Chemistry

The geometries of the target compounds were built with standard bond lengths and angles using the PC Spartan 06 program (Wavefunction, Inc.: Irvine, Ca, USA, 2006) [62]; in this sense, a conformational study was accomplished to obtain minimum energy structures using the AM1 all-valence electron self-consistent field molecular orbital approximation [63]. It is important to note that the program gives a set of 100 conformers, from which the most representative conformers are selected according to their spatial distribution and energy. The re-optimization calculations were carried out using the Gaussian 16 package (Gaussian, Inc.: Wallingford, CT, USA, 2016) [64] at the Density Functional Theory (DFT) level of theory. For DFT calculations, Becke’s three-parameter hybrid functional, B3LYP, which includes a mixture of Hartree–Fock exchange and DFT exchange-correlation [65,66], was used. The basis set employed includes the split-valence basis set 6-311++G(d,p) [61,62,63,64,65,66,67,68,69,70,71]. This last basis set contains polarized d and p, and s and p diffuse functions on carbon and oxygen atoms, which are necessary to properly describe the electronic structure of ionic states. In all cases, default convergence criteria were used.

In this work, the more relevant electronic properties [72] for stereoisomers **2**–**3**, **5**–**6** have been calculated, including electronic chemical potential (µ), ionization potential (IP), electron affinity (EA), and electronegativity (Χ) [73,74,75,76,77], as well as the energy of the highest occupied molecular orbital (HOMO) and the lowest unoccupied molecular orbital (LUMO) [78]. Using chemical potential µ and hardness η, [73] have defined another quantum chemical descriptor known as electrophilicity index (ω) (or more accurately called the global electrophilicity) [76,77], which measures the propensity to absorb electrons. That is, the electrophilicity index measures energy stabilization when an optimal electronic charge transfer from the environment to the system occurs. Physicochemical properties, which give a measure of stability and reactivity of molecules, were estimated from the calculated B3LYP/6-311++G(d,p) neutral, anion, and cation states through finite-difference expressions [9].

IP = Ecation − Eneutral(1)

EA = Eneutral − Eanion(2)

η = (IP − AE)/2(3)

µ = −(IP + AE)/2(4)

Χ = (IP + AE)/2(5)

ω = (µ^2^/2 η) (6)

Natural population analysis (NPA) defines atomic orbitals based on the molecular wave function, thereby obtaining different atomic orbitals, depending on the chemical environment [79]. The most employed property is the molecular electrostatic potential (MEP). The MEP has been used primarily for predicting sites and relative reactivity towards electrophilic or nucleophilic attack and in studies of biological recognition [80]. A red zone represents large negative values of the potential, while blue zones represent large positive values (orange, yellow, and green represent intermediate values of the potential).

### 3.2. Biological Activity Prediction and Molecular Docking

Biological activity prediction was performed employing chemoinformatic online tools, including SWISSADME (version 2017) [38], molInspiration (version 2016) [39], and Osiris Toxicity Explorer (version 2001) [43], from which physicochemical properties such as molecular weight, logP, hydrogen bond donors and acceptors, and topological polar surface area were obtained, as an approach to the Lipinski rule of five. PASSOnline (version 2.0) [45,46] was used for the compounds’ biological potential prediction.

To understand the binding mode of **2**, **3**, **5**, and **6**, docking studies were performed, employing olaparib and naproxen as controls. The complete optimization of the three-dimensional structures was achieved using the Gaussian 16 program [60] at the theory B3LYP/6311++G(d,p) level [68,69,81]. The PARP-1 catalytic domain and COX-2 crystal were retrieved from the Protein Data Bank (PDB ID 1UK0 and 3NT1), maintaining only one monomer. Since it was reported that both crystals have high stability, the structures obtained from PDB were employed to perform docking studies [53,80,82].

Docking studies were performed employing AutoDock 1.5.7 software due to its high correlation with experimental data. AutoDock Tools 1.5.7 software was retrieved from MGL tools (version 1.5.7) [83] to prepare pdbqt files of protein structures and ligands for docking. A text file containing all information about the protein and ligand pdbqt files, as well as grid box information, was created. Both the protein and the ligand were treated as rigid during the docking procedure. A directed docking was performed employing a quadratic lattice with points separated by 0.375 Å that was centered on the active site of PARP-1, and the grid center was designated at dimensions (x, y, and z): 5.092, −6.651, and 31.313 Å for PARP-1 and −40.628, −49.725, and −23.152 Å for COX-2. The binding poses with the lowest free energy binding (ΔG) were analyzed employing AutoDock Tools, and the images were created using BIOVIA Discovery Studio Visualizer 2019 [84].

### 3.3. In Vitro Citotoxicity

In vitro cytotoxicity of the compounds was evaluated in five cell lines: MDA MB 231 (epithelial adenocarcinoma of the mammary gland), MCF-7 (ductal carcinoma of the mammary gland), U373 (astrocytoma-derived glioblastoma), A549 (lung carcinoma), and U87 (human glioblastoma) (Glendale, AZ, USA, Corning 009017). These cells were cultured in DMEM F-12 medium supplemented with Glutamax (10565018, GIBCO, Waltham, MA, USA) and an antibiotic and antimycotic (100X, 15240062, GIBCO), supplemented with 10% fetal bovine serum (FBS) (16000044, GIBCO). They were incubated at 37 °C in a 5% CO_2_ atmosphere and 95% humidity. The cells were detached from the dish using trypsin/0.25% EDTA (25200072, GIBCO) at 37 °C. The reaction was stopped with DMEM F-12 medium containing 10% FBS and centrifuged at 2500 rpm for 10 min. The medium was decanted and resuspended in DMEM F-12 medium with 10% FBS. Cells were plated onto a 96-well ELISA plate and incubated again until 80% confluent. Compounds **2**, **3**, and **5** were used at a concentration of 25 μM by dissolving in FBS-free DMEM F-12 with 5% glycerol. 100 μL of the dissolved compounds was added to each well of the ELISA plate, while control wells used only DMEM F-12 without FBS and were incubated for 24 h.

At the end of the incubation, 20 microliters of MTT solution dissolved in PBS at 5 mg/mL were added and maintained under incubation conditions for 4 h. At the end of this time, the culture medium containing the MTT was decanted, and 50 microliters of isopropyl alcohol containing 4 micromolar HCl was added to dissolve the formazan formed. The mixture was stirred for 15 min and read at 540 nm in a Varioskan™ LUX multimode ELISA reader (Thermo Fisher Scientific, Waltham, MA, USA). The survival percentages obtained were calculated and compared with the control groups [56].

## 4. Conclusions

In the present study, a set of four perezone and isoperezone derivatives was studied in silico, chemoinformatically, and by molecular coupling analysis to determine their potential inhibitory activity on COX-2 and PARP-1. Also, theoretical studies were carried out employing the B3LYP hybrid functional and the 6-311++G(d,p) set of base functions to obtain electrostatic potential maps, atomic charges, and molecular orbital distribution to determine the sites that could interact with amino acid residues from both proteins. The results showed that the most stable energy interaction was accomplished with molecules **2** and **3**; additionally, **2** and **6** exhibited a selective interaction with the COX-2 isoform. On the other hand, derivative **2** was the only molecule that showed one relevant interaction with the PARP-1 catalytic triad. In compliance with reactivity properties and frontier orbitals, compounds **2** and **6** were the most reactive molecules, slightly improving their affinity towards COX-2 and PARP-1. However, the general structure of pipitzols and isopipitzols decreases the possible active interaction sites when compared to previously reported perezone.

Finally, in vitro testing with relevant cancer cell lines indicated that compound **5** exhibited the lowest IC_50_ for MDA-MB-231, MCF-7, U737, and U87, while compound **3** achieved a better IC_50_ for the A549 cell line. In this research, differences in structural, reactivity, and affinity properties between isomers were shown, opening the possibility of the study of natural and synthetic products with structural modifications with improved properties to be prospective antineoplastic candidates, having studied proteins related to cell proliferation and anti-inflammatory processes involved in cancer disease.

## Figures and Tables

**Table 1:** Electronic energy for **2**, **3**, and **5**, **6**.

Molecule	Electronic Energy (Hartrees)	Electronic Energy (kcal/mol)	Relative Energy (kcal/mol)
**2**	−809.53	−507,990.37	0
**3**	−809.53	−507,989.47	0.90
**5**	−809.51	−507,975.32	15.05
**6**	−809.51	−507,975.16	15.21

Compound **2** achieved the most stable energy optimization; it was employed for relative energy calculations.

**Table 2:** Comparison of HOMO, LUMO, and energy GAP (E_GAP_) (E_LUMO_-E_HOMO_) for molecules **2**, **3**, and **5**, **6**.

Molecule	HOMO (eV)	LUMO (eV)	ΔGAP (eV)
**2**	−6.57	−1.89	4.68
**3**	−6.56	−1.87	4.69
**5**	−6.91	−1.75	5.17
**6**	−6.63	−2.05	4.59

**Table 3:** Chemical parameters as indicators of reactivity behavior for molecules **2**, **3**, and **5**, **6**.

Molecule	Neutral	Positive ^1^	Negative ^1^	IP (eV) ^2^	EA (eV) ^3^	η (eV) ^4^	µ (eV) ^5^	Χ (eV) ^6^	ω (eV) ^7^
**2**	−809.5340	−809.2293	−809.5413	8.29	0.20	4.05	−4.25	4.25	2.23
**3**	−809.5326	−809.2365	−809.5504	8.06	0.48	3.79	−4.27	4.27	2.41
**5**	−809.5097	−809.1906	−809.5095	8.68	−0.01	4.34	−4.34	4.34	2.17
**6**	−809.5228	−809.2148	−809.5384	8.38	0.42	3.98	−4.40	4.40	2.17

^1^ Theoretical positive and negative energies correspond to the cation and anion state, respectively, at B3LYP/6-311++G(d,p) optimized geometry of the neutral ground state. Therefore, chemical parameters are defined as: ^2^ [IP] Ionization potential. ^3^ [EA] Electron affinity. ^4^ [η] Hardness. ^5^ [µ] Chemical potential, ^6^ [χ] Electronegativity, and ^7^ [ω] Electrophilicity index, in eV.

**Table 4:** Lipinski rule of 5 properties for **2**, **3**, and **5**, **6**.

Molecule	2 ^1,2^	3 ^1,2^	5 ^1,2^	6 ^1,2^
Molecular weight (g/mol)	248.32	248.32	248.32	248.32
logP	2.23	2.23	2.16	2.16
nON	3	3	3	3
nOHNH	1	1	1	1
TPSA (Å)	54.37	54.37	54.37	54.37

^1,2^ Properties obtained from References [38,39].

**Table 5:** Growth inhibition (%) of human tumor cell lines for target compounds.

Compound	Tumor Cell Lines				
	MDA-MB-231	MCF-7	U373	A549	U87
**2**	112.62	96.38	92.23	101.45	90.63
**3**	104.36	96.24	87.85	64.49	91.89
**5**	98.23	87.85	83.59	103.21	90.33

## Data Availability

The data reported in this study are available upon request to mirruv@comunidad.unam.mx (R.M.R.) and nicovain@cuautitlan.unam.mx (M.I.N.-V.).

## Supplementary Materials

The following supporting information can be downloaded at: https://www.mdpi.com/article/10.3390/molecules31030469/s1, Table S1: Bond length (Å) for molecules **2**, **3**, **5**, and **6**; Table S2. Bond angles (°) for molecules **2**, **3**, **5**, and **6**; Table S3. Calculated dihedrals (°) for molecules **2**, **3**, **5**, and **6**; Table S4: Natural atomic charges in e^−^, for molecules **2**, **3**, **5**, and **6**; Table S5: Stretching vibrational frequencies and deviation percentage for the most relevant assignments; Table S6: ^1^H NMR theoretical and experimental chemical shifts (*δ*) for **2**, **3**, **5**, and **6**; Table S7: ^13^C NMR theoretical and experimental chemical shifts (*δ*) for **2**, **3**, **5**, and **6**; Table S8: Amino acid residues and ΔG values obtained by docking studies between COX-2 and PARP-1 with target compounds **2**, **3**, **5**, and **6**; Table S9. Pharmacokinetic ADME-Tox properties prediction for compounds **2**, **3**, **5**, and **6**; Table S10: (a). PASS Online prediction report for compounds **2**, **3**; (b). PASS Online prediction report for compounds **5**, **6**; S11. Cartesian coordinates from compounds **2**, **3**, **5**, and **6.**
