# Supplementary material for: α,β-Pipitzols and α,β-Isopipitzols from Natural Quinone Perezone: Quantum Chemistry, Docking, Chemoinformatic, and Pharmacological Studies
Source: Molecules. 2026 Jan 29;31(3):469. doi: 10.3390/molecules31030469 (PMC12899246; doi:10.3390/molecules31030469)
Supplement: Supplementary file 1 [file molecules-31-00469-s001.zip › molecules-4068064-supplementary.pdf]

α,β-Pipitzols and α,β-Isopipitzols from Natural Quinone Perezone: Quantum Chemistry, Docking, Chemoinformatic, and Pharmacological Studies

Adriana Lizbeth Rivera Espejel [1], Joel Martínez [1], Cristopher Williams Fuentes Cid [1], Martha E. Macías Pérez [2], Maricarmen Hernández Rodríguez [3], Alejandro Fajardo De La Rosa [4], René Miranda Ruvalcaba [1,*] and María Inés Nicolás-Vázquez [1,*]

*Correspondence should address to: mirruv@comunidad.unam.mx (R.M.R.), nicovain@cuautitlan.unam.mx (M.I.N.V.).

CONTENT

Table S1. Bond length (Å) for molecules **2**, **3**, **5**, and **6**.

Table S2. Bond angles (°) for molecules **2**, **3**, **5**, and **6**.

Table S3. Calculated dihedrals (°) for molecules **2**, **3**, **5**, and **6**.

Table S4. Natural atomic charges in e^-, for molecules **2**, **3**, **5**, and **6**.

Table S5. Stretching vibrational frequencies and deviation percentage for the most relevant assignments.

Table S6. ^{1}H NMR theoretical and experimental chemical shifts (δ) for **2**, **3**, **5**, and **6**.

Table S7. ^{13}C NMR theoretical and experimental chemical shifts (δ) for **2**, **3**, **5**, and **6**.

Table S8. Amino acid residues and ΔG values obtained by docking studies between COX-2 and PARP-1 with target compounds **2**, **3**, **5**, and **6.**

Table S9. Pharmacokinetic ADME-Tox properties prediction for compounds **2**, **3**, **5**, and **6.**

Table S10a. PASS Online prediction report for compounds **2**, **3.**

Table S10b. PASS Online prediction report for compounds **5**, **6.**

S11. Cartesian coordinates from compounds **2**, **3**, **5**, and **6**

Table S1. Bond length (Å) for molecules **2**, **3, 5**, and **6**.

	2		3			5			6	
**Bond**	**E [1] (Å)**	**T [4] (Å)**	**E [1] (Å)**	**T [4] (Å)**	**Bond**	**E [2] (Å)**	**T [4] (Å)**	**Bond**	**E [3] (Å)**	**T [4] (Å)**
C1-C2	1.548	1.574	1.579	1.579	C1-C2	-	1.620	C1-C2	1.513	1.529
C2-C3	1.576	1.577	1.557	1.573	C2-C3	-	1.597	C2-C3	-	1.346
C3-C4	1.535	1.555	1.545	1.543	C3-C4	-	1.555	C3-C4	-	1.476
C4-C5	1.514	1.539	1.509	1.537	C4-C5	-	1.544	C4-C5	1.471	1.55
C5-C6	1.538	1.538	1.512	1.541	C5-C6	-	1.565	C5-C6	1.539	1.549
C6-C7	1.560	1.539	1.560	1.551	C6-C7	1.502	1.506	C6-C7	1.503	1.547
C7-C8	1.509	1.520	1.518	1.520	C7-C8	1.329	1.348	C7-C8	1.542	1.539
C8-C9	1.474	1.477	1.474	1.482	C8-C9	1.470	1.477	C8-C9	1.505	1.549
C9-C10	1.340	1.352	1.347	1.352	C9-C1	-	1.537	C9-C10	-	1.582
C10-C1	1.510	1.517	1.513	1.517	C1-C10	1.502	1.527	C1-C10	1.564	1.583
C1-C11	1.511	1.526	1.491	1.523	C10-C11	1.497	1.510	C1-C11	1.516	1.526
C2-C14	1.527	1.541	1.521	1.540	C11-C3	-	1.558	C11-C5	1.535	1.521
C2-C15	1.530	1.534	1.528	1.534	C11-C7	-	1.508	C5-C9	1.567	1.593
C3-C7	1.572	1.590	1.572	1.593	C1-C13	-	1.524	C2-C12	-	1.499
C6-C13	1.515	1.530	1.540	1.531	C2-C14	-	1.542	C6-C13	-	1.537
C7-C11	1.524	1.545	1.557	1.539	C2-C15	-	1.535	C10-C14	-	1.535
C12-C10	1.493	1.499	1.501	1.499	C6-C12	1.497	1.536	C6-C15	-	1.539
O1-C11	1.201	1.202	1.205	1.202	C8-O3	1.357	1.359	C1-O3	1.412	1.406
O2-C8	1.214	1.224	1.223	1.223	C9-O2	1.227	1.223	C11-O2	1.200	1.205
O3-C9	1.375	1.358	1.354	1.358	C10-O1	1.212	1.204	C4-O1	1.224	1.213

[1] Experimental from Reference [1]. [2] From Reference [2]. [3] From Reference [3]. [4] Theoretical

Table S2. Bond angle (°) for molecules **2**, **3, 5**, and **6**.

	2		3		5			6		
**Angle**	**E [1] (°)**	**T [4] (°)**	**E [1] (°)**	**T [4] (°)**	**Bond**	**E [2] (°)**	**T [4] (°)**	**Bond**	**E [3] (°)**	**T [4] (°)**
C1-C2-C3	103.3	102.3	102.0	102.4	C1-C2-C3	-	105.3	C1-C2-C3	121.1	120.2
C2-C3-C4	120.2	119.8	119.2	121.2	C2-C3-C4	-	119.2	C2-C3-C4	122.5	123.3
C3-C4-C5	103.3	103.9	106.9	106.2	C3-C4-C5	-	111.5	C3-C4-C5	114.7	114.4
C4-C5-C6	104.3	104.2	104.0	103.8	C4-C5-C6	-	111.8	C4-C5-C6	119.4	120.3
C4-C3-C7	106.6	104.3	108.5	106.4	C5-C6-C7	105.6	105.7	C5-C6-C7	104.0	101.6
C5-C6-C7	102.9	102.6	101.9	101.3	C6-C7-C8	124.6	124.6	C6-C7-C8	103.4	104.7
C6-C7-C8	118.8	115.3	116.5	115.9	C7-C8-C9	121.1	121.0	C7-C8-C9	103.2	105.7
C7-C8-C9	115.0	116.3	117.6	122.4	C8-C9-C1	117.1	117.8	C8-C9-C10	120.5	118.4
C8-C9-C10	122.7	121.9	121.0	119.2	C9-C1-C12	-	110.2	C10-C1-C2	112.3	113.0
C9-C10-C1	119.6	119.4	119.5	119.2	C9-C1-C10	105.6	104.9	C9-C10-C1	102.3	102.2
C5-C6-C13	114.6	115.5	116.7	106.1	C9-C1-C2	-	110.1	C10-C1-C11	101.5	99.8
C10-C1-C11	106.3	106.7	107.4	106.1	C10-C1-C2	99.7	99.8	C1-C11-C5	104.4	106.1
C7-C6-C13	119.4	117.4	116.0	118.7	C10-C1-C12	-	114.7	C11-C1-C2	105.2	106.0
C1-C11-C7	104.3	103.7	104.7	107.4	C10-C11-C7	-	110.9	C11-C5-C4	106.2	104.6
C1-C10-C12	117.9	118.3	117.9	100.7	C10-C11-C3	-	103.8	C11-C5-C9	101.8	100.6
C2-C1-C11	101.1	100.4	100.8	111.1	C11-C10-C1	105.1	104.8	C11-C5-C6	115.2	113.1
C3-C2-C15	113.1	111.0	113.9	113.2	C11-C7-C6	112.8	113.7	C1-C2-C12	116.6	117.1
C3-C2-C14	111.0	113.5	111.1	106.8	C11-C7-C8	119.2	118.8	C3-C2-C12	-	122.7
C3-C7-C6	106.6	106.4	106.9	101.4	C11-C3-C4	-	109.8	C5-C6-C13	118.6	117.0
C3-C7-C11	114.1	101.0	120.0	106.0	C11-C3-C2	-	104.2	C7-C6-C13	-	111.0
C3-C7-C8	106.9	108.6	108.4	106.0	C13-C6-C5	110.7	112.2	C9-C5-C6	-	107.9
C3-C7-C11	101.1	101.0	99.4	101.4	C13-C6-C7	116.0	114.6	C9-C10-C14	-	111.0
C8-C7-C11	107.7	105.0	104.9	122.4	C12-C1-C2	-	116.3	C9-C10-C15	-	114.0
C12-C10-C9	122.5	122.3	122.6	118.4	C1-C2-C14	-	109.2	C1-C10-C14	-	113.4
C12-C10-C1	-	118.3	-	128.4	C1-C2-C15	-	111.6	C1-C10-C15	-	107.8
O1-C11-C1	128.5	127.5	128.6	127.3	C3-C2-C14	-	107.5	C14-C10-C15	-	108.4

O1-C11-C7	127.3	128.8	126.5	125.5	C3-C2-C15	-	115.2	O1-C4-C5	-	124.0
O2-C8-C7	124.9	124.3	121.0	118.6	C7-C11-C3	-	104.6	O1-C4-C3	-	121.6
O2-C8-C9	120.1	119.5	121.2	114.6	O1-C10-C1	126.3	127.4	O2-C11-C5	-	130.0
O3-C9-C8	116.0	115.0	117.6	123.0	O1-C10-C11	128.4	127.7	O2-C11-C1	-	123.8
O3-C9-C10	121.4	123.1	121.3	123.0	O2-C9-C8	120.0	119.1	O3-C1-C2	-	110.7
					O2-C9-C1	122.7	123.0	O3-C1-C10	-	114. 6
					O3-C8-C7	121.7	123.2			

[1] Experimental. From Reference [1]. [2] From Reference [2]. [3] From Reference [3]. [4] Theoretical

Table S3. Calculated dihedrals (°) for molecules **2**, **3**, **5**, and **6**.

Angle	2		3		Angle	5	Angle	6
	Degrees (°)					Degrees (°)		Degrees (°)
	E [1,2]	T [3]	E [1,2]	T [3]		T [3]		T [3]
C1-C2-C3-C4	-	-133.7	-	134.6	C1-C2-C3-C4	121.3	C1-C2-C3-C4	-2.3
C2-C3-C4-C5	-	106.3	-	-149.8	C2-C3-C4-C5	-144.6	C2-C3-C4-C5	7.8
C3-C4-C5-C6	44.2	34.0	34.6	42.9	C3-C4-C5-C6	63.6	C3-C4-C5-C6	-170.3
C4-C5-C6-C7	-32.7	-41.0	-42.7	-38.6	C4-C5-C6-C7	-29.3	C4-C5-C6-C7	153.1
C5-C6-C7-C3	16.3	18.67	16.3	19.99	C5-C6-C7-C8	120.9	C5-C6-C7-C8	39.9
C5-C6-C7-C8	-	153.0	-	-100.0	C6-C7-C8-C9	-154.1	C6-C7-C8-C9	-37.6
C6-C7-C3-C4	-	-12.08	-	5.6	C8-C9-C1-C2	68.1	C8-C9-C10-C1	132.6
C7-C3-C4-C5	-32.7	-30.7	-12.3	-29.3	C9-C1-C2-C3	-82.1	C9-C10-C1-C2	74.0
C8-C9-C10-C1	-	1.0	-	-0.5	C4-C3-C11-C10	-154.8	C9-C5-C4-C3	64.9
C1-C11-C7-C6	-	157.0	-	-154.6	C13-C1-C10-C11	-170.5	C5-C11-C1-C10	50.1
C2-C1-C11-C7	-	-51.6	-	50.2	C13-C1-C2-C15	-82.7	C11-C5-C4-C3	-41.9
C7-C11-C1-C10	-	69.7	-	-70.4	C13-C1-C2-C3	151.7	C11-C1-C2-C3	31.5
O1-C11-C1-C10	-	-112.5	-	112.8	C14-C2-C3-C4	-122.4	C1-C11-C5-C6	-154.4
O1-C11-C1-C2	-	126.2	-	-126.6	O1-C10-C1-C9	-114.7	O1-C4-C5-C11	138.9
O1-C11-C7-C3	-	-136.9	-	137.3	O1-C10-C1-C13	6.3	O1-C4-C5-C6	10.4
O2-C8-C9-O3	-	-1.7	-	-1.2	O2-C11-C1-O3	-4.9	O2-C9-C8-C7	176.2

O2-C8-C9-C10	-	177.2	-	178.4	O2-C11-C5-C4	-110.7	O2-C9-C1-C10	146.6
O3-C9-C10-C12	-	2.1	-	-2.3	O3-C1-C2-C3	153.2	O3-C8-C9-O2	4.6

[1] Experimental. [2] From Reference [1]. [3] Theoretical.

Table S4. Natural atomic charges in e$^-$, for molecules **2**, **3, 5**, and **6**.

Atom	2 [1]	Atom	3 [1]	Atom	5 [1]	Atom	6 [1]
C1	-0.327	C1	-0.324	C1	-0.214	C1	0.209
C2	-0.056	C2	-0.057	C2	-0.034	C2	-0.123
C3	-0.183	C3	-0.182	C3	-0.186	C3	-0.345
C4	-0.383	C4	-0.381	C4	-0.390	C4	0.397
C5	-0.374	C5	-0.369	C5	-0.369	C5	-0.195
C6	-0.217	C6	-0.215	C6	-0.248	C6	-0.196
C7	-0.217	C7	-0.215	C7	0.034	C7	-0.373
C8	0.533	C8	0.532	C8	0.205	C8	-0.387
C9	0.209	C9	0.209	C9	0.529	C9	-0.194
C10	0.011	C10	0.009	C10	0.631	C10	-0.026
C11	0.644	C11	0.643	C11	-0.343	C11	0.591
C12	-0.603	C12	-0.602	C12	-0.560	C12	-0.570
C13	-0.573	C13	-0.573	C13	-0.581	C13	-0.566
C14	-0.580	C14	-0.581	C14	-0.579	C14	-0.575
C15	-0.572	C15	-0.571	C15	-0.587	C15	-0.590
O1	-0.532	O1	-0.534	O1	-0.681	O1	-0.701
O2	-0.590	O2	-0.591	O2	-0.588	O2	-0.641
O3	-0.681	O3	-0.681	O3	-0.529	O3	-0.768

[1] Theoretical values

Table S5. Stretching vibrational frequencies and deviation percentage for the most relevant assignments.

Molecule	Assignment	Experimental frequency (cm^{-1}) [1]	Theoretical frequency (cm^{-1}) [2]	Deviation (%)
**2**	OH	3450	3546.0	2.7963
	Cyclopentanone	1760	1763.5	0.2018
	6 members enolized α-diketone	1670	1661.8	-0.4384
		1635	1618.3	-0.7071
**3**	OH	3450	3525.9	2.4885
	Cyclopentanone	1760	1764.6	0.2662
	6 members enolized α-diketone	1670	1664.6	-0.3225
		1635	1614.1	-0.6775
**5**	OH	3464	3354.5	2.3378
	Cyclopentanone	1756	1764.1	0.4687
	6 members enolized α-diketone	1676	1667.7	-0.4871
		1632	1622.2	-0.5993
**6**	OH	-	3580.4	-
	Cyclopentanone	-	1691.8	-
	6 members enolized α-diketone	-	1759.5	-
		-	1609.7	-

[1] From References [4,5]. [2] Corrected theoretical vibrational frequencies.

Table S6. ^{1}H NMR theoretical and experimental chemical shift (δ) for **2**, **3**, **5**, and **6**.

**2**			**3**			**5**			**6**	
					**δ (ppm)**					
**Assignment**	**E** [1,2]	**T** [3]	**Assignment**	**E** [1,2]	**T** [3]	**Assignment**	**E** [4]	**T** [3]	**Assignment**	**T** [3]
O3-H	6.12	6.08	O3-H	6.1	6.15	O3-H	5.98	5.83	C3-H	6.1
C12-Me	2.83	2.93	C12-HMe [5]	2.8	2.93	C6-H	3.17	3.34	C6-H	2.9
C6-H	2.76	2.69	C1-H	2.6	2.56	C11-H	3.12	2.88	C12-HMe [5]	2.6
C1-H	2.42	2.47	C6-H	2.2	2.37	C3-H	2.43	2.24	C13-HMe [5]	2.5
C13-Me	2.17	1.96	C13-HMe [5]	2.1	2.17	C5-Heq [6]	1.92	1.85	O3-H	2.5
C12-Me	2.14	1.78	C5-H	2.0	1.93	C4-Heq [6]	1.60	1.44	C12-HMe [5]	2.1
C5-H	2.11	1.74	C3-H	2.0	1.86	C5-Hax [7]	1.30	1.36	C9-H	2.1
C12-H	2.06	1.73	C5-H	1.8	1.85	C4-Hax [7]	1.30	1.06	C8-H	1.8
C4-H	1.89	1.72	C12-HMe [5]	1.8	1.79	C12-HMe [5]	1.18	1.27	C12-HMe [5]	1.7
C3-H	1.76	1.70	C12-HMe [5]	1.7	1.77	C12-HMe [5]	1.18	1.22	C8-H	1.6
C4-H	1.65	1.58	C4-H	1.6	1.66	C12-HMe [5]	1.18	1.09	C7-H	1.5
C5-H	1.39	1.45	C4-H	1.4	1.44	C13-HMe [5]	1.17	1.23	C15-HMe [5]	1.4
C13-HMe [5]	1.37	1.31	C13-HMe [5]	1.4	1.19	C13-HMe [5]	1.17	1.20	C7-H	1.3
C15-HMe [5]	1.34	1.11	C15-HMe [5]	1.3	1.08	C13-HMe [5]	1.17	0.85	C14-HMe [5]	1.2
C15-HMe [5]	1.32	1.07	C14-HMe [5]	1.3	1.05	C14-HMe [5]	0.96	0.91	C13-HMe [5]	1.0
C14-HMe [5]	1.08	1.02	C14-HMe [5]	1.3	1.04	C14-HMe [5]	0.96	0.87	C14-HMe [5]	1.0
C14-HMe [5]	1.06	1.02	C13-HMe [5]	1.1	1.04	C14-HMe [5]	0.96	0.85	C14-HMe [5]	0.9
C13-HMe [5]	1.03	0.98	C14-HMe [5]	1.1	0.96	C15-HMe [5]	0.81	1.05	C15-HMe [5]	0.7
C14-HMe [5]	0.87	0.89	C15-HMe [5]	0.9	0.92	C15-HMe [5]	0.81	0.64	C13-HMe [5]	0.7
C15-HMe [5]	0.83	0.86	C15-HMe [5]	0.9	0.91	C15-HMe [5]	0.81	0.50	C14-HMe [5]	0.7

[1] Experimental. [2] From Reference [5]. [3] Theoretical. [4] From Reference [6]. [5] HMe (methyl hydrogen). [6] Heq (equatorial). [7] Hax (axial).

Table S7. ^{13}C NMR theoretical and experimental chemical shift (δ) for **2**, **3**, **5**, and **6**

**2**			**3**			**5**			**6**	
**δ (ppm)**										
**Assignment**	**E [1,2]**	**T [3]**	**Assignment**	**E [1,2]**	**T [3]**	**Assignment**	**E [4]**	**T [3]**	**Assignment**	**T [3]**
C11	203.6	212.4	C11	203.3	211.8	C10	206.6	211.8	C11	248.2
C8	193.7	201.4	C8	193.4	202.9	C9	195.1	202.9	C4	232.0
C9	144.6	153.5	C9	144.6	153.8	C7	140.7	153.8	C2	206.9
C10	127.2	139.5	C10	129.0	138.5	C8	143.6	138.5	C3	166.4
C7	75.0	84.2	C7	76.2	82.7	C1	70.5	82.7	C1	123.9
C1	69.0	74.9	C1	68.9	76.1	C3	47.9	76.1	C5	113.3
C3	57.4	62.6	C3	55.5	65.7	C2	44.9	65.7	C9	92.2
C2	37.3	46.1	C2	37.4	45.4	C11	46.8	45.4	C10	82.8
C6	34.9	42.6	C5	35.2	43.5	C6	32.4	43.5	C7	72.7
C5	38.2	39.5	C6	35.0	42.6	C5	32.2	42.6	C6	71.0
C4	26.0	29.2	C4	24.7	31.5	C14	29.1	31.5	C8	60.0
C14	25.5	28.7	C14	26.5	28.4	C4	21.6	28.4	C14	59.0
C15	14.9	26.1	C15	24.0	27.8	C15	21.0	27.8	C12	55.3
C12	17.8	19.9	C12	17.9	19.9	C13	16.6	19.8	C15	51.6
C13	24.4	14.9	C13	13.4	16.7	C12	8.0	16.6	C13	49.9

[1] Experimental. [2] From Reference [5]. [3] Theoretical. [4] From Reference [6].

Table S8. Amino acid residues and ΔG values obtained by docking studies between COX-2 and PARP-1 with target compounds **2**, **3**, **5**, and **6.**

Molecule	ΔG (kcal/mol) with COX-2	Amino acids' interaction with COX-2 [1]	ΔG (kcal/mol) with PARP-1	Amino acids' interaction with PARP-1 [1,2]
**2**	-7.19	**VAL523**, ALA527	-7.56	**TYR235**, SER203
**3**	-7.19	VAL349, LEU352, ALA527, **SER530**	-7.29	No interaction with the catalytic triad
**5**	-7.07	LEU352, VAL349, LEU352, **SER530**, **TYR385**	-7.95	No interaction with the catalytic triad
**6**	-6.72	**VAL523**, LEU352	-6.39	No interaction with the catalytic triad
Naproxen	-8.75	**VAL523**, LEU531, TRP387, VAL349, LEU352, **TY355**, **ARG120**	-	-
Olaparib	-	-	-10.23	**TYR235**, **HIS201**

[1] From Reference [7]. The COX-2 active site is specific regarding COX-1. [1,2] From Reference [7,8]. Main amino acid residues in the pocket site are shown in bold.

Table S9. Pharmacokinetic ADME-Tox prediction for compounds **2**, **3**, **5**, and **6.**

**Molecule**	**2**	**3**	**5**	**6**
Gastrointestinal absorption	High	High	High	High
Blood-brain barrier permeant	Yes	Yes	Yes	Yes
P-gp substrate	No	No	Yes	Yes
CYP1A2 inhibitor	No	No	No	No
CYP2C19 inhibitor	No	No	No	No
CYP2C9 inhibitor	Yes	Yes	No	No
CYP2D6 inhibitor	No	No	No	No
CYP3A4 inhibitor	No	No	No	No
Mutagenic	No	No	No	No
Tumorigenic	No	No	No	No
Irritant	No	No	No	No
Reproductive effect	No	No	No	No
Drug score	0.583	0.583	0.547	0.547

Pharmacokinetic properties obtained by the SwissADME predictor [9]. Estimated Drug score and Toxicity by Osiris Property Explorer [10].

Table S10a. PASS Online prediction report for compounds **2**, **3.**

	**2, 3**	
**Pa**	**Pi**	**Activity**
0.844	0.001	Polarisant stimulant
0.850	0.014	Testosterone 17-beta-dehydrogenase inhibitor
0.823	0.004	Cardiovascular analeptic
0.821	0.016	CYP2J substrate
0.819	0.026	Ubiquinol-cytocrome-c reductase inhibitor
0.805	0.018	Antiseborrheic
0.766	0.002	Neutrophic factor
0.748	0.020	Acylcarnitine hydrolase inhibitor
0.721	0.002	Myc inhibitor
0.704	0.002	Alpha-pinene-oxide decyclase inhibitor
0.749	0.048	CYP2C12 substrate
0.700	0.007	Carminative
0.713	0.041	Actieczematic
0.701	0.031	Alkenylglycerophosphocholine hydrolase inhibitor
0.689	0.022	Alkylacetylglycerophosphate inhibitor
0.697	0.034	CYP2J2 substrate
0.687	0.028	Antineoplasic
0.671	0.019	Phosphatase inhibitor
0.656	0.025	Antidyskinetic
0.635	0.016	Vasoprotector

Table S10b. PASS Online prediction report for compounds **5**, **6.**

	**5, 6**	
**Pa**	**Pi**	**Activity**
0.912	0.003	Cardiovascular analeptic
0.879	0.009	Testosterone 17-beta-dehydrogenase inhibitor
0.828	0.015	CYP2J substrate
0.824	0.015	Antiseborrheic
0.796	0.014	Acylcarnitine hydrolase inhibitor
0.773	0.022	Alkenylglycerophosphocholine hydrolase inhibitor
0.755	0.007	Vasoprotector
0.780	0.040	Ubiquinol-cytocrome-c reductase inhibitor
0.748	0.014	Alkylacetylglycerophosphate inhibitor
0.750	0.022	CYP2J2 substrate
0.757	0.046	CYP2C12 substrate
0.730	0.027	Membrane permeability inhibitor

0.727	0.037	Actieczematic
0.673	0.003	Postmenopausal disorders treatment
0.677	0.018	Phosphatase inhibitor
0.620	0.005	Indanol dehydrogenase inhibitor
0.624	0.010	Carminative
0.628	0.027	JAK2 expression inhibitor
0.607	0.014	Dextranase inhibitor
0.601	0.010	Dolichyl-diphospooligosaccharide-protein glycotransferase inhibitor

S11. Cartesian coordinates of optimized for **2**, **3**, **5**, and **6.**

2

C -0.21401700 -1.56629000 -0.50763900

C -1.64284500 -1.28936200 -0.25358700

C 0.76738400 -0.49154100 -0.07067700

C -2.05090000 -0.18079600 0.40486300

C 0.56254900 0.78671300 -0.99386100

H 0.06122600 0.48626400 -1.91569700

C -1.01930600 0.82377400 0.88147600

H -1.41895800 1.39074600 1.72405100

C -0.40994800 1.76145400 -0.22559900

O 0.10774200 -2.60862900 -1.06172000

O -2.50045400 -2.23949800 -0.70858700

H -1.94157300 -2.93199000 -1.10274500

C -3.49204900 0.07264500 0.73152300

H -4.13634300 -0.71030400 0.33364900

H -3.81997300 1.03722700 0.32928300

H -3.63072700 0.12033200 1.81790700

C -1.46304300 2.34760500 -1.17431400

H -0.97725600 2.94053300 -1.95576000

H -2.15418000 3.00866300 -0.64238500

H -2.04542000 1.56417400 -1.66478100

C 0.31650100 2.91621900 0.49038200

H 1.11175300 2.56849100 1.15468500

H -0.39527600 3.48147800 1.09872700

H 0.75688400 3.61232300 -0.22685000

C 0.21954200 0.02642000 1.27800000

O 0.67486000 -0.11842800 2.38043300

C 2.25374700 -0.87465600 -0.18339800

H 2.33834400 -1.42533900 -1.12825800

C 2.93844300 0.49002300 -0.37246300

H 3.95536400 0.39407100 -0.76162200

H 3.00905200 1.00637000 0.59175200

C 2.00749900 1.24806500 -1.33535500

H 2.13259800 2.33014800 -1.28236200

H 2.23524000 0.95522300 -2.36401400

C 2.83948400 -1.75820800 0.92034800

H 3.87808500 -2.00508000 0.68051000

H 2.28964700 -2.69890900 1.00292300

H 2.81608000 -1.26437000 1.89177900

3

C -0.19659000 -1.49790400 -0.36262100

C 1.28173100 -1.55409500 -0.26692600

C -0.84858800 -0.23746400 0.18205700

C 2.01066900 -0.56029800 0.28842100

C -0.48234400 0.92434100 -0.84447300

H -0.19133200 0.46740500 -1.79394800

C 1.29896500 0.66619100 0.82660600

H 1.91988000 1.15914800 1.57678400

C 0.77192100 1.67741600 -0.26535000

O -0.78385300 -2.42389700 -0.90532000

O 1.83884700 -2.67778100 -0.78746500

H 1.09307200 -3.21110100 -1.11578900

C 3.50186400 -0.63566800 0.42329800

H 3.98186100 0.22258700 -0.05910200

H 3.89756300 -1.55093400 -0.01501000

H 3.78772100 -0.60355300 1.48114000

C 1.79883100 1.97020800 -1.36662100

5

C 0.84748100 1.50931500 -0.29398500

C -0.62899400 1.49314000 -0.28264500

C 1.58131600 0.32429800 0.35267900

C -1.30883800 0.48223800 0.29473800

H 2.68619100 2.46661700 -0.96212500

H 1.36461100 2.63611800 -2.11896800

H 2.11781900 1.05713500 -1.87448200

C 0.41258000 2.99349700 0.44857900

H 1.31280000 3.43854500 0.88204700

H -0.30518600 2.85107800 1.26076400

H -0.01092000 3.71791100 -0.25148300

C -2.38707000 -0.20086900 0.37184200

H -2.54444400 0.45251600 1.23866700

C -2.87964400 0.56406600 -0.87209400

H -3.88621800 0.96970900 -0.73758100

H -2.90795500 -0.10936100 -1.73631200

C -1.81950700 1.65836500 -1.07461400

H -1.97150600 2.44663700 -0.33314900

H -1.86367400 2.12496200 -2.06194800

C -0.01672400 0.16878200 1.41109400

O -0.36343200 0.15296000 2.56188000

C -3.12269200 -1.51000100 0.66825600

H -2.69866900 -2.01170900 1.54188300

H -3.08335900 -2.20174600 -0.17199800

H -4.17116500 -1.29161600 0.89313600

C 1.34724200 -1.01909800 -0.52118300

C -0.53952100 -0.57878200 1.04136400

H -1.14638400 -1.03421000 1.82450800

C -0.07643300 -1.57747300 -0.06067300

H 0.09342000 -2.54880500 0.41283900

O 1.42755800 2.40753500 -0.88813300

O -1.22258800 2.45459400 -1.03748200

H -0.49677800 3.00172700 -1.38587300

C 0.75325900 -0.02212900 1.58800500

O 1.08665800 0.06981400 2.74141200

C 3.03084900 0.69710200 0.64156300

H 3.51838400 -0.06533800 1.24770000

H 3.07053300 1.63614700 1.19556900

H 3.59194300 0.83847900 -0.28350200

C 1.48598700 -0.74508300 -2.02492000

H 2.48380100 -0.35772200 -2.24696300

H 0.76482900 -0.01841000 -2.40143200

H 1.35684300 -1.67008800 -2.59416500

C 2.40640900 -2.06883000 -0.12914000

H 3.41263800 -1.75925500 -0.41818500

H 2.18852300 -3.00962800 -0.64313800

H 2.40804200 -2.27075700 0.94587200

C -2.70454600 0.06337100 -0.08721400

H -2.99874300 0.66214100 -0.95400800

C -1.20128300 -1.75339500 -1.12036500

H -1.18730400 -2.77559000 -1.50844000

H -1.03664000 -1.09803400 -1.97816500

C -2.59047000 -1.43381900 -0.52803500

H -2.78170600 -2.08489100 0.33282600

H -3.37373500 -1.65196800 -1.25928200

C -3.75583300 0.26433700 1.01499300

H -3.48241000 -0.26707000 1.93158100

H -4.72744200 -0.11633100 0.68650000

H -3.87059800 1.32291600 1.25828800

6

C -1.69247400 1.66255400 -1.00989800

C -0.51845000 0.65352100 -0.96974800

C -0.95239700 -0.39032000 0.15204900

C -2.39384300 -0.03837900 0.59670900

C -2.44262600 1.48333800 0.32164800

H -1.36740000 2.68982700 -1.17742000

H -2.35303600 1.40371600 -1.84123400

H -3.46764300 1.85967900 0.27984000

H -1.93282600 2.02681600 1.12267400

C -3.54305400 -0.76515000 -0.11925200

H -3.54955600 -1.83016600 0.09671600

H -4.49106400 -0.33195600 0.21423400

H -3.49062500 -0.65674600 -1.20599000

H -2.47679000 -0.23313600 1.66942800

C 0.08899100 -0.10081700 1.22251500

O -0.03724600 0.00640300 2.41647200

C 1.40266800 0.13253100 0.48292500

C 0.92243900 1.18894400 -0.59384900

C 0.91970400 2.57576900 0.07436700

H 1.92749600 2.81615300 0.41307100

H 0.60673800 3.34403300 -0.63528000

H 0.26033400 2.62995600 0.94384000

C 1.82603500 1.24928100 -1.83270300
H 1.89379800 0.28233500 -2.33634400
H 1.42389100 1.96938600 -2.55185600
H 2.83549100 1.57574400 -1.56772900
O 2.41485000 0.59373200 1.34306700
H 2.02164500 0.61165500 2.22982000
C -0.61382700 -1.81792000 -0.34676500
O -1.44612300 -2.66527700 -0.59282000
C 0.82867000 -2.07598600 -0.52286800
H 1.07806200 -3.04033700 -0.95243300
C 1.78572800 -1.21400900 -0.13208300
C 3.24731000 -1.53271000 -0.22247900
H 3.68895800 -1.54314300 0.77656300
H 3.40868100 -2.49968600 -0.70037300
H 3.78359300 -0.76152100 -0.78280000
H -0.44084400 0.14464800 -1.93188300

References

1. Evans, S.V.; Yee, V. C.; Garcia-Garibay, M.; Trotter, J. Structure of a Mixed Crystal of α- and β-Pipitzol (1:1). *Struct. Commun.* **1994**, *50* (2), 278-281, https://doi.org/10.1107/S0108270193009060.
2. Hernández-Ortega S.; Yuste F.; Ortiz, B.; Barrios, H.; Sánchez-Obregón R.; Walls, F. β-Isopipitzol. *Acta Crystallogr. Sect. C*: *Cryst. Struct. Commun.* **1996**, *52* (6), 1452-1454, https://doi.org/10.1107/S0108270195015988.
3. Huipe-Nava E.; Mendoza V.; García E.; Guzmán, J. A.; Salvador, J. L.; Soriano-García M. Structure of α-Isopipitzol (4,8,8,10-Tetramethyl-9-Hydroxy-2,12-Dioxotricyclo[5,3,1,03,7]Undec-1-En). *Anal. Sci.* **2000**, *16* (11), 1239-1240, https://doi.org/10.2116/analsci.16.1239.
4. Walls, P.; Padilla, J.; Joseph-Nathan, P.; Giral, F.; Romo, J. The structures of α and β-pipitzols. *Tetrahedron Lett.* **1965**, *21*, 1577-1582, https://doi.org/10.1016/S0040-4039(01)84094-6.
5. Joseph-Nathan, P.; Gutiérrez, A.; Hernández, J. D.; Román, L. U.; Santillán, R. L. 13C-Nmr Studies of Cedranolides. *J. Nat. Prod.* **1986**, *49* (1), 79-89, https://doi.org/10.1021/np50043a008.
6. Yuste, F.; Barrios, H.; Díaz, E.; Ortiz, B.; Sánchez-Obregón, R.; Walls, F. The Structure of β Isopipitzol. *Tetrahedron Lett.* **1994**, *35* (50), 9329-9332, https://doi.org/10.1016/S0040-4039(01)84094-6.
7. Ahmadi, M.; Bekeschus, S.; Weltmann, K.-D.; von Woedtke, T.; Wende, K. Non-Steroidal Anti-Inflammatory Drugs: Recent Advances in the Use of Synthetic COX-2 Inhibitors. *RSC Med. Chem.* **2022**, *13* (5), 471-496, https://doi.org/10.1039/d1md00280e.
8. Rubiales-Martínez, A.; Martínez, J.; Mera-Jiménez, E.; Pérez-Flores, J.; Téllez-Isaías, G.; Miranda Ruvalcaba, R.; Hernández-Rodríguez, M.; Mancilla Percino, T.; Macías Pérez, M. E.; Nicolás-Vázquez, M. I. Design of Two New Sulfur Derivatives of Perezone: In Silico Study Simulation Targeting PARP-1 and in Vitro Study Validation Using Cancer Cell Lines. *Int. J. Mol. Sci.* **2024**, *25* (2), 868. https://doi.org/10.3390/ijms25020868.
9. SwissADME: a free web tool to evaluate pharmacokinetics, drug-likeness, and medicinal chemistry friendliness of small molecules. *Sci. Rep*. 2017, *7*, 42717.
10. Sander, T., OSIRIS Property Explorer. Organic Chemistry Portal, **2001**, https://www.organic-chemistry.org/prog/peo.
